# Acute gout attack in a peritoneal dialysis patient treated with Firsekibart: a case report

**DOI:** 10.1515/med-2026-1454

**Published:** 2026-06-03

**Authors:** Wenqian Wei, Lijie Gu, Hanyu Meng, Nan Zhu, Shu Rong

**Affiliations:** Department of Nephrology, Shanghai General Hospital, Shanghai Jiaotong University School of Medicine, Shanghai, China

**Keywords:** gout, Firsekibart, IL-1β, peritoneal dialysis, case report

## Abstract

**Objectives:**

Managing acute gout attacks in renal failure patients on peritoneal dialysis (PD) is challenging. We report a unique case of a PD patient with recurrent pulmonary infections, heart failure, and severe anemia, who presented with refractory gout attacks successfully treated with the IL-1β monoclonal antibody Firsekibart.

**Case presentation:**

A 52-year-old female PD patient experienced recurrent swelling and pain in limb joints for one year, with acute exacerbation for three days. Laboratory tests showed serum uric acid (UA) 627 μmol/L and C-reactive protein (CRP) 16.6 mg/L. Conventional treatments with nonsteroidal anti-inflammatory drugs (NSAIDs) and glucocorticoids were ineffective. Given the patient’s high inflammatory state and multiple comorbidities, Firsekibart (200 mg) was administered. Joint pain and swelling significantly improved within 24 h. The Visual Analogue Scale (VAS) score decreased from 5 to 1 within 72 h, and CRP dropped from 16.6 mg/L to 6.5 mg/L at day 3. UA decreased from 485 μmol/L to 388.9 μmol/L at day 7. At three-month follow-up, the patient remained free of gout recurrence, with VAS 1, CRP 7.8 mg/L, and UA 393 μmol/L. At the last 7-month telephone follow-up she reported no further acute gout attacks, though mild joint swelling persisted. No injection site reactions, hypersensitivity, or infectious complications were observed throughout the entire follow-up period.

**Conclusions:**

IL-1β blockade Firsekibart may represent a safe and effective option for managing acute gout attacks in high-risk ESRD patients on PD with multiple comorbidities.

## Introduction

Patients with chronic kidney disease (CKD), particularly those with end-stage renal disease (ESRD) undergoing maintenance dialysis, commonly exhibit a persistent, non-infectious, low-grade systemic inflammatory response known as the “microinflammatory state.” This condition contributes to malnutrition, anemia, and renal fibrosis, and serves as an independent risk factor for cardiovascular events and mortality [1]. Within the complex cytokine network mediating this state, interleukin-1β (IL-1β) occupies an upstream position in the inflammatory cascade – a mechanism particularly relevant for ESRD patients with hyperuricemia and gout, who often exhibit elevated IL-1βlevels.

However, anti-inflammatory treatment of acute gout attacks in ESRD patients presents significant challenges. Nonsteroidal anti-inflammatory drugs (NSAIDs), colchicine, and glucocorticoids each have limitations and safety concerns in this population [2]. Interleukin-1 (IL-1) inhibitors target the NOD-like receptor family pyrin domain containing 3 inflammasome pathway, blocking IL-1β effects on cells and exerting anti-inflammatory actions in affected joints [3], potentially offering a therapeutic option for acute gout in advanced CKD patients [4]. Firsekibart, a recombinant fully human anti-IL-1β monoclonal antibody, specifically neutralizes IL-1β, thereby disrupting the vicious cycle of gout and systemic inflammation. Phase III clinical studies have demonstrated that Firsekibart is safe and effective for acute gout attacks in patients with estimated glomerular filtration rate >30 mL/min/1.73 m^2^, reducing the risk of gout recurrence at 12 and 24 weeks by 90 % and 87 %, respectively [5].

Here, we report a case of acute gout attack in a maintenance peritoneal dialysis patient with multiple comorbidities, in whom conventional anti-inflammatory therapy failed, resulting in persistently elevated inflammatory markers and recurrent gout flares. Treatment with Firsekibart achieved a significant therapeutic response, providing a potential reference for managing gout attacks in patients with ESRD.

## Case report

### Patient information and clinical presentation

A 52-year-old female on maintenance peritoneal dialysis (PD) for over two years presented on September 6, 2025, with recurrent bilateral upper limb joint swelling and pain for one year, acutely exacerbated for three days. She had a five-year history of hypertension.

Her renal history began in 2020 when elevated serum creatinine was incidentally detected. In September 2022, she was admitted with fatigue and chest tightness; laboratory tests revealed serum creatinine 1,212.7 μmol/L and potassium 6.91 mmol/L. She was diagnosed with stage 5 CKD and commenced on maintenance PD. Over time, due to the patient’s gradual increase in serum creatinine levels, poor volume control, as well as recurrent pulmonary infections and acute exacerbations of heart failure, the peritoneal dialysis regimen was continuously adjusted based on changes in her condition, and intermittent hemodialysis was added in September 2024. Current PD regimen: 2.5 % low-calcium PD solution 2 L × 5 bags combined with intermittent hemodialysis.

### Gout history and previous treatments

In May 2024, the patient developed acute pain in the right upper limb, hands, and feet, suspected as gout. Glucocorticoids provided temporary relief. By September 2024, she experienced severe right shoulder and elbow pain with subcutaneous masses over the left elbow and thumb, and swelling of distal interphalangeal joints. Attacks increased in frequency, involving multiple joints with tophi formation.

Treatment with NSAIDs, glucocorticoids, and antibiotics – alone or in combination – proved ineffective. Febuxostat was initiated but discontinued due to desquamative pain; serum uric acid (UA) fluctuated between 470 and 580 μmol/L. Symptoms persisted despite hemodialysis.

Diagnostic challenge: Differentiating septic arthritis from gout was difficult given recurrent infections, but tophi, persistent hyperuricemia, and antibiotic non-response supported acute gout.

### This event

**Figure 1:**
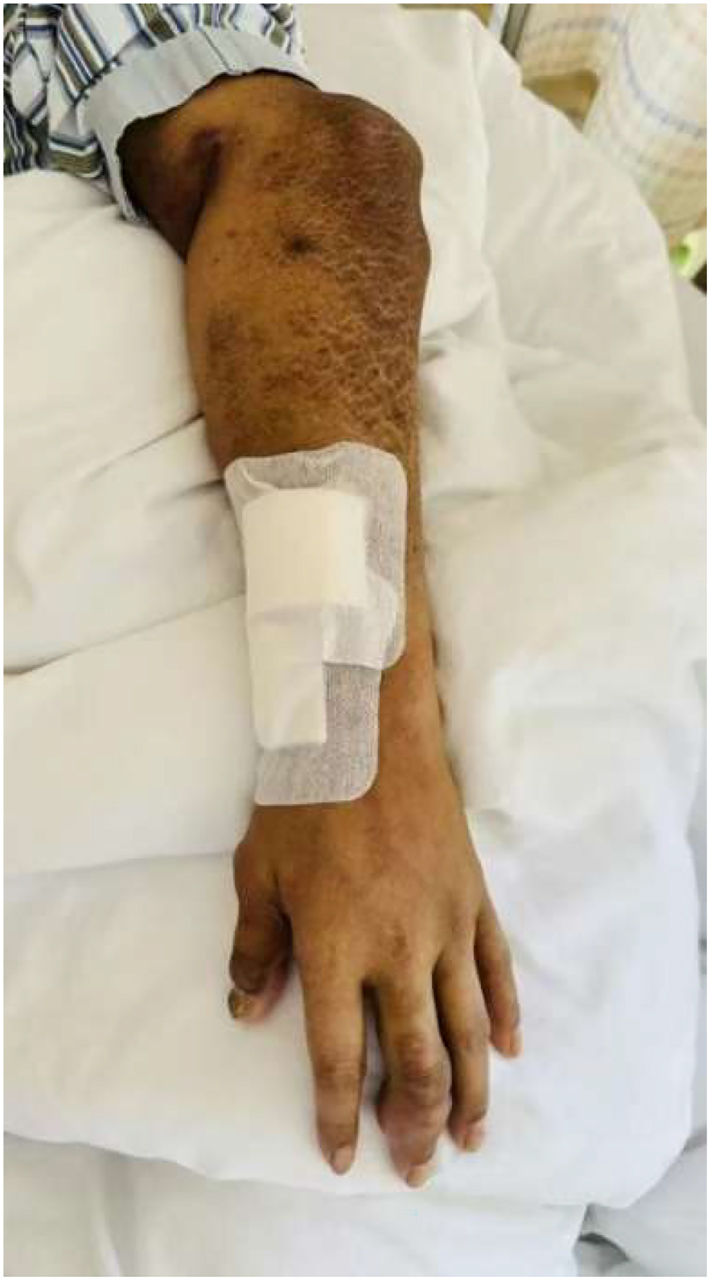
Swelling of the left elbow and interphalangeal joints with visible tophi.

**Figure 2:**
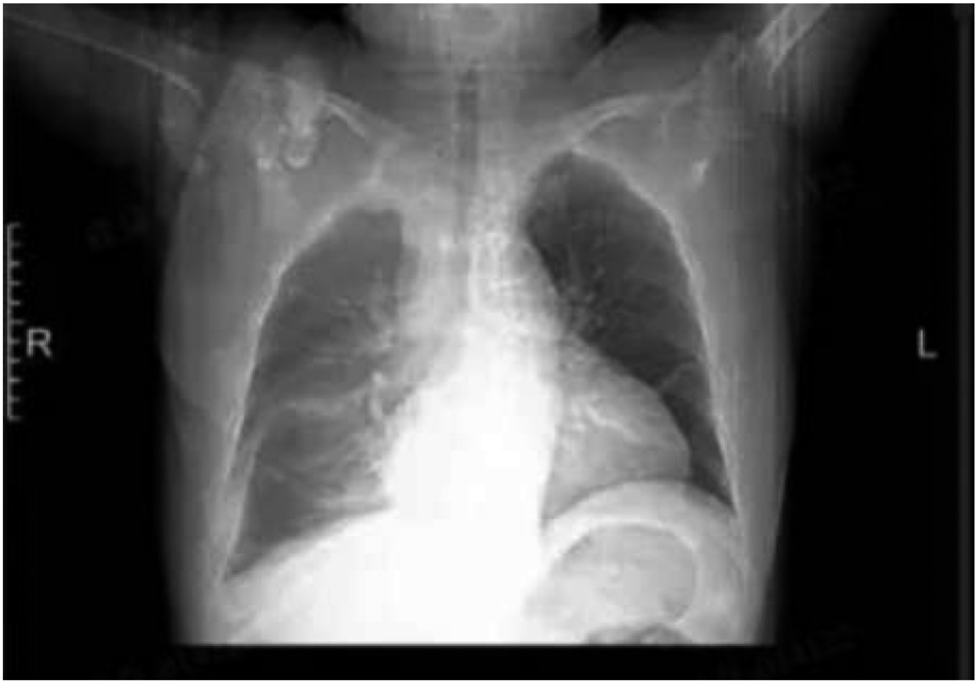
Chest CT showing pleural effusion and cardiomegaly.

In September 2025, the patient again experienced swelling and pain in both hand joints (Figure 1). Chest CT showed pleural effusion and cardiomegaly (Figure 2). UA was 627 μmol/L, CRP was 16.6 mg/L, and PCT was 0.3. After admission, treatment with glucocorticoids and NSAIDs for 3 days was ineffective. Considering the patient’s recurrent gout attacks, obvious tophi, poor response to conventional anti-inflammatory treatment, poor peritoneal dialysis efficiency, poor baseline cardiopulmonary function, long-term chronic inflammation, and high risk of cardiovascular events, after comprehensive evaluation, on September 10, 2025, the patient received a subcutaneous injection of 200 mg of IL-1β monoclonal antibody Firsekibart.

### Timeline of this patient

1) 2020: First detected elevated creatinine (asymptomatic).
2) September 2022: Diagnosed with CKD stage 5; initiated PD (2L × 3 bags/day).
3) April 2024: Pulmonary infection + acute heart failure; intensified PD regimen.
4) May 2024: First gout attack; treated with glucocorticoids.
5) September 2024: Recurrent gout with tophi formation; hemodialysis added.
6) September 6, 2025: Current admission with acute gout flare.
7) September 10, 2025: Firsekibart 200 mg subcutaneous administration.
8) September 13, 2025: Day 3 follow-up: VAS 1, CRP 6.5 mg/L.
9) September 17, 2025: Day 7 follow-up: VAS 1, CRP 2.1 mg/L, UA 388.9 μmol/L.
10) December 16, 2025: Month 3 follow-up: VAS 1, CRP 7.8 mg/L, UA 393 μmol/L, PCT 0.24 ng/mL.

## Clinical follow-up and outcome

### Short-term efficacy

The response to Firsekibart was rapid and significant (Table 1). At day 7 follow-up, joint swelling had completely resolved with only mild residual soreness that did not affect daily activities. The patient subsequently commenced febuxostat 20 mg daily for urate-lowering therapy.

**Table 1:** Patient’s response to Firsekibart.

Time point	VAS score	CRP, mg/L	UA, μmol/L	PCT	Clinical status
Baseline (Day 0)	5	16.6	485	0.30	Severe joint swelling and pain
Day 1	3	/	/	/	Noticeable improvement within 24 h
Day 3	1	6.5	/	/	Significant pain relief, reduced swelling
Day 7	1	2.1	388.9	0.23	Joint swelling resolved, mild soreness only
Month 3	1	7.8	393	0.24	No recurrence; mild residual swelling without pain/redness

### Mid-term follow-up (December 16, 2025)

The patient was hospitalized for a peritoneal equilibration test. Physical examination revealed persistent mild joint swelling without erythema or tenderness. She self-reported no further gout attacks since discharge. Laboratory tests showed UA 393 μmol/L, CRP 7.8 mg/L, and PCT 0.24 ng/mL. The VAS score remained 1.

### Long-term telephone follow-up (April 23, 2026)

The patient reported that no acute gout attacks had occurred since the previous follow-up. However, mild joint swelling persisted. She continued oral febuxostat and maintained disease control with combined peritoneal dialysis and intermittent hemodialysis.

### Safety and tolerability

The single-dose injection was well-tolerated. No injection site reactions, hypersensitivity, or infectious complications were observed during the entire follow-up period (from September 10, 2025, to April 23, 2026). No adverse or unanticipated events were reported.

## Discussion

Patients with CKD are at high risk of gout attacks, with an overall prevalence of up to 24.3 %, of which the prevalence of hyperuricemia in patients with stage 5 CKD is as high as 35.6 % [6]. As renal function deteriorates, the frequency, severity and tophi of gout attacks increase significantly [7], affecting patients’ quality of life and increasing the medical burden. Moreover, CKD combined with gout can significantly increase the risk of patients progressing to end-stage renal disease, with a risk ratio up to 18.9 times that of people without CKD or gout [8].

For patients with CKD stage 4 and above, especially those who have entered the dialysis stage, anti-inflammatory treatment of gout attacks is challenging: there is a lack of high-quality clinical research evidence, and the efficacy and safety of traditional anti-inflammatory treatments for gout such as NSAIDs, colchicine and glucocorticoids are uncertain, requiring close monitoring of related adverse reactions [4]. A multicenter retrospective study showed that anakinra can safely and effectively control gout attacks in patients with CKD stage 4–5 or kidney transplantation [9], suggesting that blocking the IL-1β pathway, a core inflammatory factor in gout, may be an effective means of treating gout attacks in ESRD and dialysis patients. The latest “Guidelines for Anti-inflammatory Treatment of Gout 2025” recommends that IL-1 inhibitors can be considered for acute gout attacks in patients with G4-5 CKD [10]. In addition, small sample studies have shown that IL-1 inhibitors such as canakinumab and anakinra can effectively control acute gout attacks in patients with advanced CKD without affecting renal function [9]. As a new generation of IL-1β monoclonal antibody, Firsekibart has the potential for long-term anti-inflammatory effects against gout attacks. In a phase III clinical trial, Firsekibart reduced the risk of gout recurrence at 12 and 24 weeks [5] and also significantly reduced the risk of gout attacks during UA-lowering treatment in gout patients, with better results than colchicine [11].

This case involves a long-term maintenance peritoneal dialysis patient with high inflammation and multiple comorbidities. For patients with recurrent infections, the use of immunosuppressants or biologics requires extreme caution. In this case, the patient was initiated with Firsekibart during a period of stable infection control. The single-dose injection was well-tolerated, with no adverse reactions or infectious complications observed during follow-up through April 2026, supporting its short-term safety in this vulnerable population. This case report shows that advanced CKD, especially in patients on maintenance dialysis, often presents with multiple complications, weak cardiopulmonary function, and a chronic inflammatory state, making conventional anti-inflammatory treatments often ineffective. Firsekibart can rapidly relieve joint swelling and pain and control gout attacks, making it a new option for gout attacks in patients with ESRD. However, its long-term efficacy and safety in the CKD gout population still require further validation through more real-world studies or clinical trials.
